# Combined wide-field optical coherence tomography angiography density map for high myopic glaucoma detection

**DOI:** 10.1038/s41598-021-01661-0

**Published:** 2021-11-11

**Authors:** Yu Jeong Kim, Kyeong Ik Na, Han Woong Lim, Mincheol Seong, Won June Lee

**Affiliations:** 1 Department of Ophthalmology, Hanyang University College of Medicine, Seoul, Korea; 2 Department of Ophthalmology, Hanyang University Seoul Hospital, 222-1, Wangsimni-ro Seongdong-gu, Seoul, 04763 Korea; 3 Department of Ophthalmology, Kangdong Sacred Heart Hospital, Hallym University College of Medicine, Seoul, Korea; 4 Department of Ophthalmology, Hanyang University Guri Hospital, Guri, Korea

**Keywords:** Neuroscience, Visual system, Glaucoma, Optic nerve diseases, Diagnostic markers

## Abstract

The present study aimed to evaluate the diagnostic ability of wide-field optical coherence tomography angiography (OCTA) density map for detection of glaucomatous damage in high myopic (HM) eyes and to further compare the diagnostic ability of OCTA with that of conventional imaging approaches including red-free photography and swept-source OCT (SS-OCT) wide-field maps. A total of 77 healthy HM eyes and 72 HM eyes with open angle glaucoma (OAG) participated in this retrospective observational study. Patients underwent a comprehensive ocular examination, including wide-field SS-OCT scan and peripapillary area and macular OCTA scans. An integrated OCTA density map thereafter was merged by vascular landmark-guided superimposition of peripapillary and macular superficial vascular density maps onto the red-free photography (resulting in the OCTA-PanoMap). Glaucoma specialists then determined the presence of glaucomatous damage in HM eyes by reading the OCTA-PanoMap and compared its sensitivity and specificity with those of conventional images. Sensitivity and specificity of OCTA-PanoMap for HM-OAG diagnosis was 94.4% and 96.1%, respectively. Compared with other imaging methods, the sensitivity of OCTA-PanoMap was significantly higher than that of red-free photography (P = 0.022) and comparable to that of wide-field SS-OCT maps. Specificity of OCTA-PanoMap was significantly higher than those of other conventional imaging methods (except for wide-field thickness map). The OCTA-PanoMap showed good diagnostic ability for discrimination of HM-OAG eyes from healthy HM eyes. As a complementary method of an alternative imaging modality, OCTA-PanoMap can be a useful tool for detection of HM-OAG.

## Introduction

Myopia has been considered as an independent risk factor for glaucoma, and moderately to highly myopic (HM) eyes have a higher risk for developing this condition than low myopic or hyperopic eyes do^1–3^. The prevalence of myopia has been increasing dramatically, especially among East Asians^4,5^. Therefore, diagnosis and monitoring glaucoma in myopic eyes are becoming very important; however, it is challenging to diagnose this condition, especially in HM eyes^6^. The reasons for the difficult diagnosis of glaucoma in HM include optic nerve head tilt, increased ovality/rotation/torsion, larger peripapillary atrophy^7–9^, and the temporal shift of Bruch’s membrane opening^10^ and the retinal nerve fiber layer (RNFL) peaks^11,12^.

Various imaging modalities are used for evaluating the glaucomatous structural damages in HM eyes. Due to structural distortion of the optic disc in these eyes (including disc tilt or large peripapillary atrophy), evaluation of the optic disc through the stereoscopic photographs is very difficult. Further, the visibility of the RNFL is poor in HM eyes on red-free photography. Optical coherence tomography (OCT) is used as a relatively objective imaging method^13^; however, image analysis is not easy due to the absence of normative database for HM.

There have been many attempts to overcome these difficulties when using OCT. The macular parameters (ganglion cell complex)^14^, its asymmetry analysis^15^, and asymmetrical differences across the horizontal raphe^16^ have shown improved diagnostic power or been adjunctive diagnostic tools for glaucoma in HM eyes. Some studies suggested approaches to improve the diagnostic power by modifying the RNFL temporalization or magnification by using the self-constructing myopic normative database in peripapillary^17,18^ or macular area^19^. Recently, Kim et al. reported that wide-field thickness and deviation map of swept-source OCT (SS-OCT) exhibited better accuracy and diagnostic power for detection of glaucomatous defects in myopic eyes than the conventional maps from spectral domain OCT did^20^.

OCT angiography (OCTA) is a relatively new imaging modality that enables us to noninvasively assess the peripapillary and macular microvasculature. Studies have shown that OCTA is useful for detecting and quantifying glaucomatous damage and its progression. Peripapillary retinal vessel density is lower in glaucomatous eyes than in healthy eyes, and the decreased vessel density in OCTA images shows an exact topographical correlation with the RNFL defect^21^. The location of focal perfusion loss in peripapillary retina showed good agreement with visual field defect^22^. There are many studies on the association between peripapillary choroidal microvascular dropout (MVD), which is visible in the deeper layer of OCTA, and glaucoma^23–27^. There are also studies on changes in the macular OCTA in glaucomatous eyes. Furthermore, many OCTA studies have been conducted on HM eyes, the relationship between MVD and healthy HM^28^ eyes and HM glaucoma^29^, and the diagnostic abilities of several superficial vessel density parameters^30^.

Recently, many studies have attempted to integrate the information of peripapillary and macular areas analysis together in the field of glaucoma^31–37^. Nowadays, with the development of OCT for diagnosing and monitoring glaucoma, it is becoming more common to analyze the optic nerve and the macular area together (both in software and hardware) rather than each area separately. However, there were not many studies that have incorporated this concept into OCTA and furthermore, as yet, there have been no studies using wide-field OCTA Map itself.

The purpose of the present study was to evaluate the diagnostic ability of the wide-field OCTA density map for the detection of glaucomatous damage in HM eyes and further compare the diagnostic ability of OCTA with that of conventional imaging approaches, including red-free photography and SS-OCT wide-field maps.

## Results

### Clinical demographics

Table 1 shows the clinical demographics in all patients at the time of enrollment. This retrospective cross-sectional study enrolled 149 eyes: 77 healthy HM eyes and 72 HM eyes with open-angle glaucoma (OAG). Four healthy HM eyes and five HM eyes with OAG were excluded due to poor image quality. The average age was 45.7 ± 14.4 years for HM-OAG eyes and 42.3 ± 15.8 years for healthy HM eyes (the controls) (p = 0.182). The differences in age, gender, IOP, spherical equivalent, AXL, and previous refractive or cataract surgery were not significant between the two groups. However, the MD, PSD, visual field index, peripapillary RNFL, macular ganglion cell—inner plexiform layer (GCIPL), and macular ganglion cell complex (GCC) thickness differed between the healthy HM eyes and those with HM-OAG (p < 0.001).

**Table 1:** Clinical demographic characteristics of the participants.

	High myopic OAG (N = 72)	Healthy high myopia (N = 77)	P value
Age (years)	45.7 ± 14.4	42.3 ± 15.8	0.182
Gender/female (%)	24 (33.3)	35 (45.5)	0.136
IOP (mmHg)	15.8 ± 3.5	16.1 ± 4.0	0.724
SE (Diopter)	−8.79 ± 2.81	−8.21 ± 2.47	0.273
AXL (mm)	27.46 ± 1.31	27.03 ± 1.07	0.086
Refractive surgery (%)	13 (16.9)	9 (12.5)	0.495
Cataract surgery (%)	7 (9.7)	11 (14.3)	0.457
RNFL thickness (μm)	69.3 ± 18.5	89.6 ± 17.0	< 0.001
GCC thickness (μm)	86.2 ± 12.5	103.1 ± 8.7	< 0.001
GCIPL thickness (μm)	54.9 ± 8.8	64.5 ± 5.4	< 0.001
MD (dB)	−7.40 ± 6.35	−2.47 ± 2.02	< 0.001
PSD (dB)	6.07 ± 4.02	2.32 ± 1.48	< 0.001
VFI (%)	82.7 ± 19.5	96.8 ± 3.5	< 0.001

The data are expressed as means ± standard deviation or no. (%).

Comparisons are performed using the chi-square test for categorical variables and the independent t-test for continuous variables.

*OAG* open angle glaucoma, *IOP* intraocular pressure, *SE* spherical equivalence, *AXL* axial length, *RNFL* retinal nerve fiber layer, *GCC* ganglion cell complex, *GCIPL* ganglion cell inner plexiform layer, *MD *mean deviation of visual field, *PDS* pattern standard deviation of visual field, *VFI* visual field index.

### Inter-rater agreement between two glaucoma specialists for detection of glaucomatous defect

Two independent glaucoma specialists (WJL and KIN) rated the presence of RNFL defects from red-free photography, wide-field thickness, and deviation map from SS-OCT and OCTA-PanoMap. The agreement obtained was moderate for red-free photography (Kappa = 0.596) and substantial for other wide-field maps (including OCTA-PanoMap) from SS-OCT (Kappa 0.741–0.771) between the two specialists.

### Diagnostic ability for discriminating between HM healthy eyes and HM glaucomatous eyes

**Figure 1:**
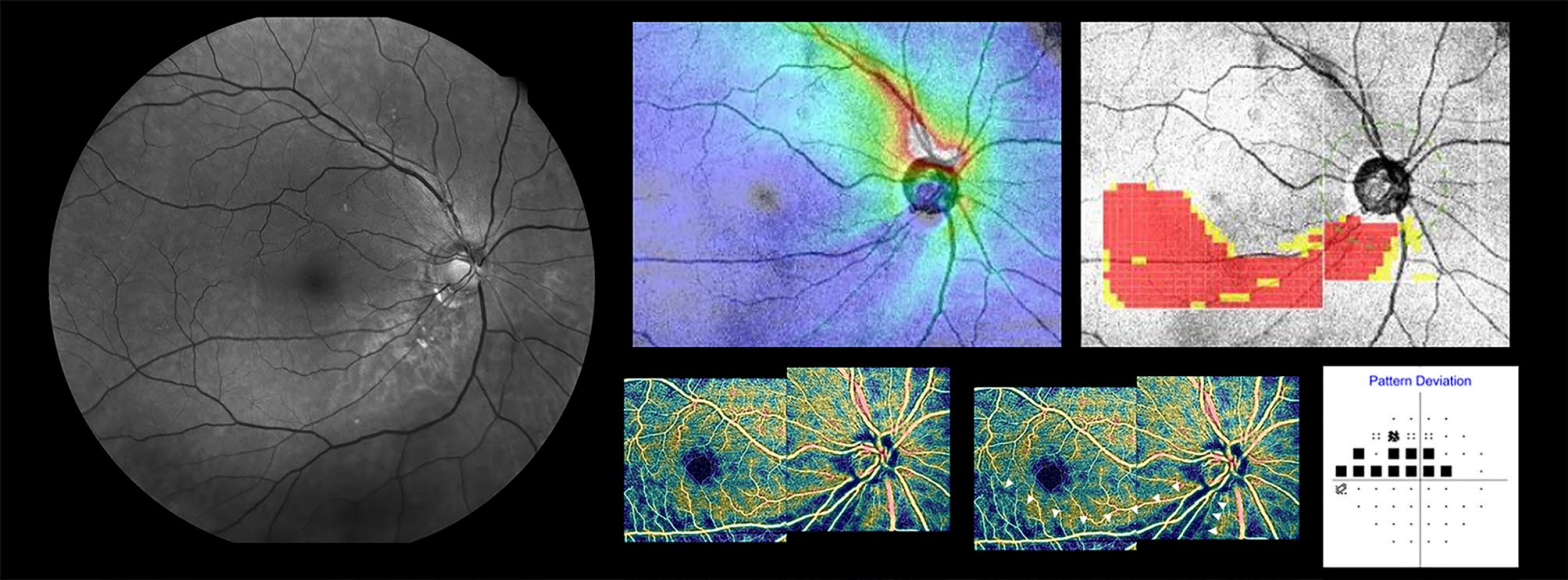
Representative case of the wide-field OCTA density map (OCTA-PanoMap) in high myopic glaucoma. Right eye of a 65-year-old female with high myopic open-angle glaucoma (axial length 27.04 mm, spherical equivalent -8.125 D). Inferotemporal RNFL defect, which is not clearly visible in red-free fundus photography is well visualized in wide-field SS-OCT thickness map, deviation map, and OCTA-PanoMap (white arrow head). Superior scotoma is confirmed using visual field tests. *OCTA* optical coherence tomography angiography, *RNFL* retinal nerve fiber layer, *SS-OCT* swept-source OCT.

**Figure 2:**
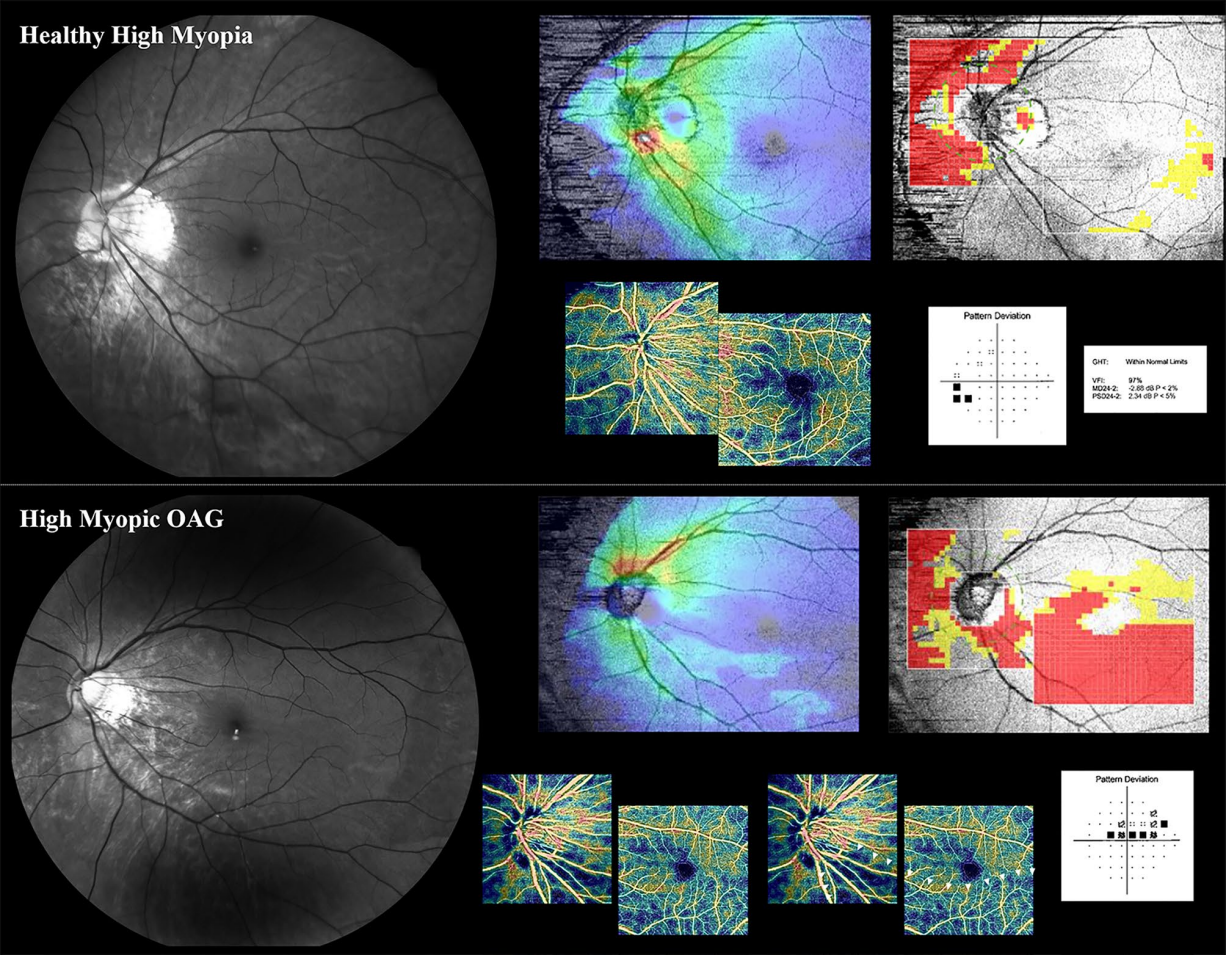
Example of highly myopic eyes in patients (upper row: male/56, −8.625 D, Axial length: 27.80 mm) (lower row: male/26, axial length: 27.86 mm, s/p LASEK). In the left eye of lower row, inferotemporal defect in vessel density is visible in the OCTA-PanoMap and consistent with the superior defect on the HVF pattern deviation map. Therefore, the diagnosis of glaucoma is made. In the upper row, however, any attenuation in vessel density is not visible in the OCTA-PanoMap and consistent with the absence of scotoma on the HVF pattern deviation map. Many red and yellow pixels (false positives) appear in the wide-field deviation map on that eye. *HVF* Humphrey visual fields, *OCTA* optical coherence tomography angiography.

The sensitivity and specificity of the categorical variables including OCTA-PanoMap, red-free photography, wide-field RNFL thickness map, wide-field deviation map (according to manual rating and criteria), and peripapillary RNFL, GCIPL, and GCC thicknesses using the abnormal criteria (< 5% level or < 1% level) provided by the built-in normative database for discriminating between HM healthy and HM-OAG eyes are presented in Table 2. Sensitivity and specificity of OCTA-PanoMap was 94.4% and 96.1%, respectively. Compared with other categorical variables, the sensitivity of OCTA-PanoMap was significantly higher than that of the red-free photography (p = 0.022). Other variables sensitivities showed no significant difference compared to that of OCTA-PanoMap. Among the categorical variables, the two highest specificity values were those for the OCTA-PanoMap (96.1%) and the wide-field RNFL thickness map (97.4%). Specificity of OCTA-PanoMap was significantly higher than those of other categorical variables (except for those of wide-field RNFL thickness map). Representative cases showing usefulness OCTA-PanoMap in detection of HM glaucomatous defect are provided in Figs. 1 and 2.

**Table 2:** Sensitivities and specificities for discrimination between high myopic open angle glaucoma and healthy high myopia.

	Sensitivity	P-value	Specificity	P-value
OCTA-PanoMap	94.4%		96.1%	
Red-free RNFL photography	81.9%	**0.022**	85.7%	**0.039**
Wide-field RNFL thickness map	84.7%	0.065	97.4%	1.000
Wide-field deviation map (manual)	97.2%	0.687	77.9%	**0.001**
Wide-field deviation map (criteria)	100.0%	NA	9.1%	** < 0.001**
**Peripapillary RNFL thickness**				
Quadrant < 5% (yellow)	93.1%	1.000	23.4%	** < 0.001**
Quadrant < 1% (red)	86.1%	0.146	48.1%	** < 0.001**
Clock hours < 5% (yellow)	98.6%	0.250	18.2%	** < 0.001**
Clock hours < 1% (red)	91.7%	0.727	40.3%	** < 0.001**
**Macular parameters**				
GCC < 5% (yellow)	95.8%	1.000	57.1%	** < 0.001**
GCC < 1% (red)	91.7%	0.727	76.6%	** < 0.001**
GCIPL < 5% (yellow)	98.6%	0.250	42.9%	** < 0.001**
GCIPL < 1% (red)	90.3%	0.453	70.1%	** < 0.001**

To qualify as “yellow” or “red” was at least 1 sector for the analysis being yellow or red.

P value index compared with OCTA-PanoMap (McNemar’s test).

Bold values indicate statistical significance with p-value less than 0.05.

*OCT-A* optical coherence tomography—angiography, *RNFL* retinal nerve fiber layer, *GCC* ganglion cell complex, *GCIPL* ganglion cell inner plexiform layer.

**Figure 3:**
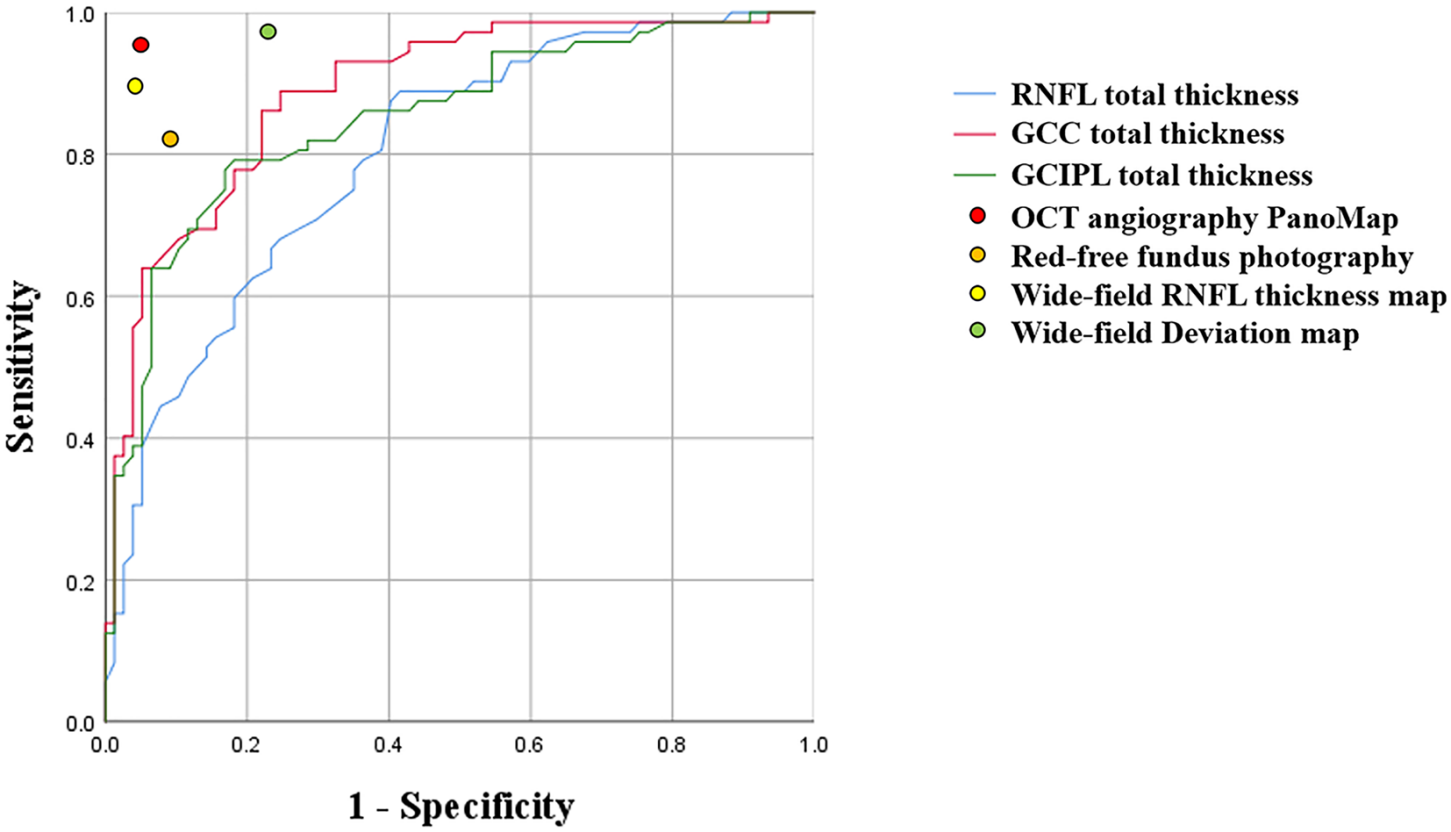
Comparison of the area under the receiver operating characteristic curves (AUCs) for discriminating between high myopic open angle glaucoma and healthy high myopia. AUC value of retinal nerve fiber layer (RNFL), ganglion cell complex (GCC), and ganglion cell-inner plexiform layer (GCIPL) thicknesses was 0.800, 0.889 and 0.852, respectively.

The AUCs in terms of quantitative measurement are presented in Fig. 3. AUC values were 0.800, 0.889 and 0.852 for peripapillary RNFL, GCC and GCIPL thicknesses, respectively. The diagnostic ability of categorical variables was also plotted on the graph as a point.

## Discussion

The present study compared diagnostic accuracy for the detection of glaucomatous defects in HM eyes between wide-field OCTA density map (OCTA-PanoMap) and conventional red-free photography or wide-field maps from SS-OCT (Topcon DRI OCT). Data demonstrated the clinical utility of OCTA-PanoMap for the diagnosis of HM-OAG.

OCTA has been widely studied and used in recent years in the diagnosis of glaucoma over the peripapillary and macular areas and imaging of the superficial and deep layers.

OCTA demonstrated reproducible focal loss of the radial peripapillary capillary (RPC) in patients with early OAG compared with that in normal controls^21,38–40^. Using OCTA, reduced peripapillary retinal perfusion in glaucomatous eyes can be visualized as focal defects and quantified as peripapillary flow index and vessel density^40^. Significant reductions in the RPC density were correlated with the site of RNFL decrease and visual field loss in glaucoma^21,40,41^. There was also an attempt to build a normative database based on the change in the RPC density with age and to create a deviation map using this database^42^.

This decrease in the superficial blood vessel density in glaucoma has also been reported in the macular area. Most studies have shown decreased perfusion in the superficial vascular complex of the macular area in patients with glaucoma using OCTA and that these decreases were correlated with visual field loss^43–47^.

Recently, many studies have attempted to integrate the information of peripapillary and macular areas using OCT in the field of glaucoma^31–37^. With the development of OCT, it is becoming more common to analyze these two areas together rather than individually. Wide-field OCT scan and map from SS-OCT and the integrated map (PanoMap) of SD-OCT are representative examples of areas currently being commercialized. These may help to well visualize continuous glaucomatous structural damage topographically and well facilitate the understanding of the spatial relationship between two areas in glaucoma^37,48^. Research on applying this concept to OCTA is limited, and a few study with similar attempts are introduced below.

Wide-field montage OCTA can visualize the expansion of the RPC network. The RPC density was also correlated well with the RNFL thickness^49^. The wide-field OCTA (8 × 8 mm) scan can visualize RPC information in a large area using software montage, but it can only cover the peripapillary area^50^. These studies attempted to reveal wider application areas using OCTA, but all were confined to the peripapillary area and did not address glaucoma^51^.

One study showed that significant microvascular damage was present in both the macular and peripapillary areas in early OAG. This study used vessel density parameters of OCTA in both the areas but evaluated only quantitative parameters, not those in HM glaucoma^52^. OCTA measurements detect changes in retinal microvasculature (in the density of both the peripapillary and macular vessels) before visual field damage is detectable in patients with OAG^53^. Another study also compared the vessel density in each of the two areas among the control, OAG, and angle closure glaucoma groups^54^. Those studies evaluated the vessel density in both the areas, but all analyses were focused on parameters and not on topographic analysis with the map.

There have also been many OCTA studies related to glaucoma in myopia and HM. OCTA measurements can be useful for diagnosing glaucoma in HM eyes, especially when using calculated indices such as macular vessel density ratio (outer macular vessel density/inner macular vessel density)^30^. Some studies compared the vascular–function with structure–function correlations in HM glaucomatous eyes. Peripapillary vessel density, as measured using OCTA, may be useful in the evaluation of glaucomatous visual field damage in HM eyes. The performance of the vessel density was remarkable even when the RNFL measured using OCT was not reliable due to segmentation errors^55,56^.

The present study has several differences from existing OCTA studies. First, a topographic map was used, not the parameters of OCTA. Second, two topographic maps of the optic nerve and macula were combined and analyzed. Finally, this method was applied to HM to compare the diagnostic ability of detecting glaucoma using conventional imaging methods.

Because this study was only performed on HM eyes, the inter-grader agreement between glaucoma specialists in this study was not higher than that reported in other studies performed with the general population. This is because it is difficult to diagnose glaucoma itself in HM, and in the process of determining any factor that could lead to its diagnosis, the method used in this study (OCTA-PanoMap) was devised.

The OCTA-PanoMap proposed in this study has two main strengths. First, the OCTA-PanoMap can visualize the structural glaucomatous damage more objectively in HM. The sensitivity of the OCTA-PanoMap was significantly superior to that of red-free photography, and the specificity of the OCTA-PanoMap was significantly superior to that of other OCT categorical parameters. As mentioned earlier, the presence of the tigroid fundus poses a significant challenge in the evaluation of glaucomatous damage in HM. Although OCT affords reproducible measurement of the RNFL thickness (swept-source technology was used in this study), the detection of RNFL abnormalities in HM eyes is complicated because of the high rate of false-positive errors (especially in OCT deviation map), which is likely related to the lack of inclusion of individuals with HM in the normative database. Second, the OCTA-PanoMap could visualize the RNFL defect at a glance in HM, making it much easier to apply clinically than when using quantified data. Maps such as thickness and deviation maps are more intuitive data than the numbers and can help in actual practice. As the images of the two areas are combined and shown as one, the continuous decrease in the capillary plexus from the optic nerve to the macular is well visualized.

This imaging method still has some limitations. First, until now, the scan range was not wide due to hardware limitations, and even though wide-field OCTA has been developed to capture a wide area, it took a long time to scan, and it was difficult to obtain a good quality image. Second, a tool for quantitative analysis of OCTA is still lacking, and only a qualitative analysis method was used in this study. Thus, the development of a more objective method is needed. Third, although this method can play an important role in imaging, it only plays a limited role in revealing the pathophysiology. There are debates as to whether the changes seen in the OCTA-PanoMap are primary or secondary. It is assumed that axon damage occurs first by glaucoma, and subsequently, the blood vessels in that area are reduced. Finally, topical medication might influence OCT parameters, including vessel density, and need to be taken into consideration^57,58^.

In conclusion, the wide-field OCTA density map (OCTA-PanoMap) showed good diagnostic ability for the discrimination of HM glaucoma from healthy HM eyes. As a complementary method of an alternative imaging modality, the OCTA-PanoMap of SS-OCT can serve as a useful tool for the detection of HM glaucoma.

## Methods

The study protocol was approved by the Institutional Review Board (IRB) of Hanyang University Hospital. Informed consent was waived due to its retrospective nature and it was confirmed by IRB of our institution. The study design adhered to the tenets of the Declaration of Helsinki for biomedical research.

### Participants

This retrospective cross-sectional study enrolled patients with healthy HM eyes and HM eyes with OAG who visited the Glaucoma Clinic of Hanyang University Hospital between August 2018 and December 2020.

All participants underwent complete ophthalmologic examination, including visual acuity testing, manifest refraction assessment, slit-lamp examination, intraocular pressure (IOP) measurements using Goldmann applanation tonometry, gonioscopy, dilated fundus examination, axial length measurement (IOLMaster; Carl Zeiss Meditec, Dublin, CA, USA), stereo disc photography, red-free photography (EIDON confocal scanner; CenterVue, Padua, Italy), 24–2 perimetry using the Swedish interactive thresholding algorithm (Humphrey Field Analyzer II; Carl Zeiss Meditec, Jena, Germany), and SS-OCT (DRI-OCT Triton; Topcon, Tokyo, Japan).

A visual field was considered to be reliable if the fixation losses were < 20%, the false positive rate was < 15%, and the false-negative rate was < 15%. A normal visual field was defined as that with a mean deviation (MD) and pattern standard deviation (PSD) within 95% confidence limits, and results of the glaucoma hemifield test (GHT) within normal limits. Eyes with glaucomatous visual field defects were defined as those with a cluster of three points with probabilities of < 5% on the pattern deviation map in at least one hemifield, including at least one point with a probability of < 1%; or a cluster of two points with a probability of < 1%, and a GHT result outside 99% of the age-specific normal limits or a PSD outside 95% of the normal limits. The visual field defects were confirmed using two consecutive reliable tests^59^.

The inclusion criteria were as follows: best-corrected visual acuity of 20/40 or higher, HM, and an open anterior chamber angle. HM was defined as refraction less than −6.0D or Axial length (AXL) > 26 mm. The exclusion criteria were as follows: history of ophthalmic surgery (e.g., glaucoma filtration surgery), severe glaucoma with an MD worse than −20 dB, any other ocular disease that could interfere with the visual function, any media opacity that could significantly interfere with OCT acquisition, and an inability to obtain a high-quality OCT image. All of the OCT scan images had to have an image quality score ≥ 30, according to the manufacturer’s recommendation^20,29^. Eyes with poor OCTA images were excluded on the basis of the following criteria^53^: image quality < 30, poor-clarity images, localized weak signal caused by artifacts such as floaters, residual motion artifacts visible as irregular vessel patterns or disc boundary on the en face angiogram or segmentation failure. One eye was randomly chosen for the study in cases where both the eyes met all the eligibility criteria.

We identified patients with OAG using several features in addition to an open angle confirmed using gonioscopy. The first feature was the presence of a characteristic optic disc with localized or diffuse neuroretinal rim thinning, increased cupping, or a cup-to-disc ratio difference > 0.2 between the eyes observed in the stereo disc photograph. The presence of an RNFL defect on red-free fundus imaging was an alternative feature. The presence of glaucomatous visual field defects was a prerequisite for glaucoma diagnosis, and patients with preperimetric glaucoma (normal visual field) were excluded from this study. A patient diagnosed with OAG was treated with topical medication or not according to the decision of the glaucoma specialist. Healthy eyes were defined as those without any history or evidence of intraocular surgery, intraocular pressure of ≤ 21 mmHg with no history of increased intraocular pressure, absence of glaucomatous appearance of the optic disc, and normal ophthalmologic findings. The wide-field OCT scan analysis was performed according to the right-eye orientation. A senior glaucoma specialist (MS) determined whether the participants were HM-OAG or HM healthy controls. All previous medical records, including images and visual field results, were reviewed to examine disease progression and confirm the diagnosis. Detailed objective method used to determine progression is provided in the Supplementary Note.

### Swept-source optical coherence tomography

We employed a wide-field scan protocol (12 × 9 mm^2^) using the DRI-OCT device. The DRI-OCT is an SS-OCT device that uses a wavelength-sweeping laser with a central wavelength of 1050 nm and tuning range of approximately 100 nm. We acquired 100,000 A-scans per second with an 8-µm axial tissue resolution. A 12 × 9 mm^2^ scan contained 256 B-scans, each containing 512 A-scans for a total of 131,072 axial scans/volume. The wide-field RNFL thickness map and wide-field deviation map were obtained and the method has been described in detail in a previous study^32^.

### OCTA imaging

We imaged the optic disc and macular OCTA using a commercially available SS-OCTA system (DRI OCT Triton; Topcon), separately. Scans were obtained from 4.5 × 4.5 mm^2^ cubes, centered on the optic disc and macula, respectively. We used the automated layer segmentation of the OCT instrument software to generate en face images of the retinal vasculature from the selected en face slabs. The density map of the selected en face slabs were presented as a color-coded map.

### Construction of the wide-field OCTA density map

With the built-in analysis software, superficial retinal layer [from the internal limiting membrane (ILM) to the inner border of the inner nuclear layer] of the macular and the density map of radial peripapillary capillary layer (from top to 70.2 µm below ILM) were used for the present study.

We used a combined wide-field OCTA density map for single-image evaluation of glaucoma diagnosis. The map was created by the same-day superimposition of optic disc and macular OCTA density maps onto RNFL photography as aligned using the Photoshop software (Version 11.0; Adobe, San Jose, CA, USA) based on vascular landmarks. Each combined map was labeled by date, and all, as collectively integrated, were defined as the OCTA-PanoMap.

### Definition of the RNFL defect

RNFL defect on red-free photography, wide-field RNFL thickness map, and wide-field deviation map has been described in detail in a previous study^32^.

RNFL defect on red-free photography was defined as having a width at a 1-disc-diameter distance from the edge of the disc larger than that of a major retinal vessel, diverging in an arcuate or wedge shape^60,61^. RNFL defect on the wide-field RNFL thickness map was defined as an arcuate or wedge-shaped diverging dark-blue area surrounding an abrupt color scale change that appeared less thick than neighboring areas on color-coded maps of wide RNFL thickness. The minimum defect size was larger than the diameter of a major retinal vessel^31,32,62,63^. RNFL defect on the SuperPixel map was arbitrarily defined as the presence of a wedge-shaped area of at least 20 contiguous yellow/red pixels along with RNFL thinning on the deviation map^31,32,60,64^. Since there were many yellow/red pixels on the deviation map of HM patients, the judgment was made by dividing the ones judged by the glaucoma specialist (manually) and those judged by criteria (20 contiguous pixels). RNFL defect on the OCTA-PanoMap was defined similarly to that on wide-field RNFL thickness map, as an arcuate or wedge-shaped diverging dark area surrounding an abrupt color scale change that appeared less crowded than neighboring areas did on color-coded density maps.

RNFL defects on above-mentioned images were evaluated independently by two glaucoma specialists (WJL, KIN) in a masked fashion. For prevention of bias, the graders who evaluated the wide-field maps were masked to the red-free photography. When the two experts disagreed, a third ophthalmologist (MS) evaluated the photographs and the disagreement was resolved after discussion.

### Statistical analyses

Statistical tests were performed using IBM SPSS Statistics 24 (IBM Corp, Armonk, NY) and MedCalc (MedCalc Software, Ostend, Belgium). The independent t-test was used to compare the characteristics of the continuous variables and the chi-squared test was used for the categorical variables. Inter-rater agreement between two glaucoma specialists (WJL, KIN) was evaluated using the Kappa test. Cohen suggested the Kappa result be interpreted as follows: values ≤ 0 as indicating no agreement, 0.01–0.20 as none to slight, 0.21–0.40 as fair, 0.41–0.60 as moderate, 0.61–0.80 as substantial, and 0.81–1.00 as almost perfect agreement^65^. To assess the abilities of each categorical variable, such as wide-field RNFL, thickness map, or the criteria on the basis of the comparison of measurements with a built-in normative database to distinguish HM glaucoma from HM healthy controls, sensitivities and specificities were tested. The comparison between sensitivities or specificities were performed using the McNemar test. The areas under the receiver operating characteristic curve (AUCs) were calculated for the continuous variables and the comparison of AUC values of the parameters was assessed using the method described by DeLong et al.^66^. P values < 0.05 were considered statistically significant. The values are presented as the mean ± standard deviation.

## Supplementary Information

Supplementary Information.
