# Supplementary material for: Combined wide-field optical coherence tomography angiography density map for high myopic glaucoma detection
Source: Sci Rep. 2021 Nov 11;11:22034. doi: 10.1038/s41598-021-01661-0 (PMC8585951; doi:10.1038/s41598-021-01661-0)
Supplement: Supplementary file 1 — Supplementary Information. [file 41598_2021_1661_MOESM1_ESM.pdf]

Supplementary Note

Determination of glaucoma progression

Progressive optic disc changes (i.e., focal or diffused rim narrowing, neuroretinal rim notching, increased cup-to-disc ratio, and adjacent vasculature position shift) were determined by comparing all series of stereo-disc photographic images and indicated glaucoma progression. Changes in retinal nerve fiber layer (RNFL) defects were determined from all series of RNFL photographs and defined as the appearance of a new defect or an increase in the width or depth of an existing defect. These changes indicated a clinically confirmed structural progression.[1]

Patients were classified as having functional progression if new and reproducible glaucomatous visual field (VF) defects, corresponding to structural damage, were found on standard automated perimetry.[2] If there were enough VF tests for progression analysis, VF progression was determined by the Early Manifest Glaucoma Trial criteria or linear regression analysis of the VF index. Early Manifest Glaucoma Trial progression was confirmed when at least 3 test points were flagged as having deteriorated significantly at the same test point locations in 3 consecutive fields.[3] These changes also had to have been observed at the latest follow-up visit. In the linear regression analysis, VF progression was defined as a significant negative slope between VF index and age.

References

1. Suh MH, Kim DM, Kim YK, et al. Patterns of progression of localized retinal nerve fibre layer defect on red-free fundus photographs in normal-tension glaucoma. Eye (Lond) 2010;24(5):857-63.
2. Kim KE, Jeoung JW, Kim DM, et al. Long-term follow-up in preperimetric open-angle glaucoma: progression rates and associated factors. Am J Ophthalmol 2015;159(1):160-8.e1-2.
3. Heijl A, Leske MC, Bengtsson B, et al. Measuring visual field progression in the Early Manifest Glaucoma Trial. Acta Ophthalmol Scand 2003;81(3):286-93.
